# Evaluation of Swallowing Related Muscle Activity by Means of Concentric Ring Electrodes

**DOI:** 10.3390/s20185267

**Published:** 2020-09-15

**Authors:** Javier Garcia-Casado, Gema Prats-Boluda, Yiyao Ye-Lin, Sebastián Restrepo-Agudelo, Estefanía Perez-Giraldo, Andrés Orozco-Duque

**Affiliations:** 1 Centro de Investigación e Innovación en Bioingeniería, Universitat Politècnica de València, 46022 Valencia, Spain; gprats@ci2b.upv.es (G.P.-B.); yiye@ci2b.upv.es (Y.Y.-L.); 2 Grupo de Investigación e Innovación Biomédica, Instituto Tecnológico Metropolitano, Medellín 050012, Colombia; sebastianrestrepo133884@correo.itm.edu.co (S.R.-A.); estefaniaperez@itm.edu.co (E.P.-G.); andresorozco@itm.edu.co (A.O.-D.)

**Keywords:** concentric ring electrodes, Laplacian potential, swallowing, surface electromyography

## Abstract

Surface electromyography (sEMG) can be helpful for evaluating swallowing related muscle activity. Conventional recordings with disc electrodes suffer from significant crosstalk from adjacent muscles and electrode-to-muscle fiber orientation problems, while concentric ring electrodes (CREs) offer enhanced spatial selectivity and axial isotropy. The aim of this work was to evaluate CRE performance in sEMG recordings of the swallowing muscles. Bipolar recordings were taken from 21 healthy young volunteers when swallowing saliva, water and yogurt, first with a conventional disc and then with a CRE. The signals were characterized by the root-mean-square amplitude, signal-to-noise ratio, myopulse, zero-crossings, median frequency, bandwidth and bilateral muscle cross-correlations. The results showed that CREs have advantages in the sEMG analysis of swallowing muscles, including enhanced spatial selectivity and the associated reduction in crosstalk, the ability to pick up a wider range of EMG frequency components and easier electrode placement thanks to its radial symmetry. However, technical changes are recommended in the future to ensure that the lower CRE signal amplitude does not significantly affect its quality. CREs show great potential for improving the clinical monitoring and evaluation of swallowing muscle activity. Future work on pathological subjects will assess the possible advantages of CREs in dysphagia monitoring and diagnosis.

## 1. Introduction

Patients with dysphagia patients find it difficult or impossible to swallow food or liquids. Dysphagia affects millions of people every year (about 3 million in the United States alone [1]) and is a substantial burden on healthcare systems. Among the current methods for its diagnosis and monitoring, surface electromyography (sEMG) stands out because it is non-invasive, relatively inexpensive, it provides a considerable amount of information on the swallowing process and can potentially be used in human-computer interfaces. Although sEMG recordings from conventional disc electrodes still have limitations, these can be overcome by concentric ring electrodes (CREs). The present study aims to evaluate the use of CREs in sEMG recording of swallowing by comparing the performance of CREs with that of conventional disc electrodes.

Swallowing is a complex process that involves the sequential voluntary and involuntary contraction of 26 muscles. Swallowing disorders (dysphagia) are highly prevalent in patients with neuromuscular or neurogenic diseases, for instance, between 27 and 50% of stroke patients and 75 to 97% of those with Parkinson’s disease develop dysphagia [2,3]. It is present in between 20 and 55% of dermatomyositis cases and in 18% of those with polymyositis [4]. The prevalence of oropharyngeal dysphagia in the general population varies between 2.3 and 16% [5] and is estimated to be in 10% of the elderly population, although in care home residents this can increase to 50% [6]. Dysphagia also has a substantial health and cost burden on healthcare systems. In the US, the total cost of treating inpatients is between USD 4.3 to 7.1 billion annually in additional hospital costs [1] and hospital stays are 40.4% longer for patients with oropharyngeal dysphagia than their non-dysphagic counterparts [7].

Dysphagia is a potentially dangerous condition that can lead to various complications [8] including aspiration pneumonia, chronic respiratory disease, poor nutrition, and dehydration. Well-established and organized plans for its detection, diagnosis and treatment can significantly reduce the symptoms. In healthcare practice, the routine detection tests can be grouped into clinical (non-instrumental) and instrumental tests. The usual clinical test is the bedside swallow examination, which depends on the training and experience of the healthcare personnel who perform it. The gold standard instrumental test is videofluoroscopy, a radiographic study of modified swallowing in which the patient chews and swallows various bolus textures of liquids and solids containing barium [9]. However, its use is limited as a tool for monitoring the evolution of the disease due to the associated risk of exposure to ionizing radiation. Additionally, it does not always identify neuromuscular abnormalities during swallowing [10].

Surface electromyography (sEMG) has been proposed as a promising diagnostic tool to evaluate the physiology of swallowing [10,11]. It eliminates the risks of radiation, infection and pain involved in conventional diagnostic methods. sEMG can be used as a complementary instrumental tool for monitoring the evolution of a wide range of neurological and functional diseases [10]. sEMG signals provide quantitative electrophysiological information about swallowing and are an important source of information on the sequential activation of the muscles involved. Studies have analyzed the activity of these muscles by acquiring the sEMG from the channels associated with the masseter muscles, orbicularis oris and the groups of submental (suprahyoid) and infrahyoid muscles [12]. Previous works have shown the usefulness of sEMG recordings from the supra and infrahyoid muscles in the diagnosis of certain pathologies associated with swallowing [10].

Surface EMG activity is usually recorded by disk electrodes in a bipolar configuration. Differential bipolar recording is better able to reject common noise compared to monopolar referential recording. The bipolar configuration also has an associated spatial filtering effect that, in addition to the low-pass filtering effect of the tissue volume conductor, smooths the sEMG signal [13]. The poor spatial resolution of conventional bipolar sEMG records can lead to crosstalk from surrounding muscles [13,14]. There are several concerns with regard to the use of sEMG to evaluate swallowing, mainly because of the small size of the muscle bellies of the lower face and submental area with overlapping fibers [15], which makes it difficult to achieve enough spatial selectivity to avoid crosstalk when using conventional electrodes. The sternocleidomastoid bounds infrahyoid muscles, which are covered with platysma, which is also activated during swallowing and causes crosstalk in sEMG measurements [15,16]. Additionally, signals from the submental surface and anterior neck are difficult to register in many individuals due to increased subdermal fat in this area [16].

Concentric ring electrodes (CREs) have been proposed to improve surface bipolar recordings of bioelectric signals as they can directly obtain an estimation of surface Laplacian potential, i.e., the second spatial derivative of the bioelectric potential on the body surface. CREs are made up of a series of concentric poles of conductive material including a central disc-shaped element and one (or more) larger outer rings. CREs act as a spatial filter that diminishes the contribution of distant bioelectric dipole sources and emphasizes the closest ones [13]. They provide an enhanced ability to differentiate multiple bioelectric dipole sources and reduce mutual information [17,18]. They also avoid problems related to the orientation of the electrode on the body with respect to muscle fibers, which is an important issue in sEMG recordings due to the fact that electrode misalignment can change the amplitude and spectral content, especially when the fiber orientation is not easy to identify [19]. CREs were first used in electrocardiography to acquire high spatial resolution surface ECG signals [20]. They were later developed in different configurations [21], dimensions and materials (from rigid substrates [22] to flexible or textile ones [23]) to pick up a wide range of bioelectric signals such as electroencephalographic [22,24], electrohysterographic [25,26], electroenterographic [27,28] and diaphragmatic sEMG [29], and others from the skeletal muscles [30]. The masseter EMG has been recorded by CRE in clinical sleep bruxism (SB) diagnosis in the muscles involved in the swallowing process [31]. This setup was adopted due to its easy applicability and design and potential reduction of EMG crosstalk. The results of a portable device based on bilateral masseter sEMG recordings by CRE (and ECG) showed good agreement with those from a conventional commercial polysomnography system in terms of diagnosing SB episodes, although the sEMG signal characteristics from the different electrode types were not compared. 

Recent studies on using ultra-flexible electrodes [32] and electrode patches [33] deal mainly with measuring sEMG signals, especially in the submental area, for portable and remote monitoring of swallowing tasks and human-computer interaction, and focus on the materials and fabrication procedures; however, they do not address the optimal electrode configuration.

Thus, the aim of this study was to assess the performance of flexible disposable CREs in recording swallowing muscle myoelectric activity in the pharyngeal phase in healthy volunteers by comparing them with conventional bipolar recordings from disc electrodes to identify their possible advantages and disadvantages.

## 2. Materials and Methods

**Figure 1:**
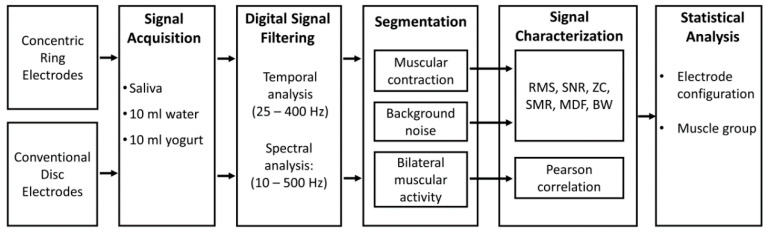
Scheme of the method used to evaluate the surface electromyography (sEMG) recording of swallowing with conventional disc electrodes (CDEs) and concentric ring electrodes (CREs). RMS: root mean square; SNR: signal-to-noise ratio; SMR: signal-to-motion ration; ZC: zero-crossing; MDF: median frequency, BW: bandwidth.

Figure 1 shows a scheme of the method followed to evaluate the sEMG recording of swallowing with CREs to compare their performance to that of conventional disc electrodes (CDEs). It comprises the signal acquisition from different muscle groups involved in the swallowing process of different liquids, signal conditioning, segmentation of sEMG intervals of interest, their characterization and comparison. A detailed description of the different steps is provided in the following subsections.

### 2.1. Study Subjects

The recordings were taken from 21 healthy young volunteers (5 males and 16 females) aged between 21 and 34 years old (24.3 ± 3.9). The exclusion criteria were: active inflammatory processes (in the mouth, head or neck), congenital oral malformations, strange elements in the mouth (like piercing), history of head or neck cancer, plastic surgery in the oral, buccal or mental regions or diagnosed cognitive disorders (motor or sensorial). The subjects were informed about the nature of the study and signed an informed consent approved by the Ethics Committee of the *Instituto Tecnológico Metropolitano* (Medellín, Colombia) by an authorization issued on 5 September 2015.

### 2.2. Signal Acquisition

The protocol used for signal acquisition was a modified version of the one proposed by Vaiman et al. [12]. Surface EMG signals were measured on both sides of three muscle groups: masseter, submental group (suprahyoid); and the infrahyoid muscles in the region of the laryngeal muscles. These groups are involved in the oral and pharyngeal swallowing phases [34].

**Figure 2:**
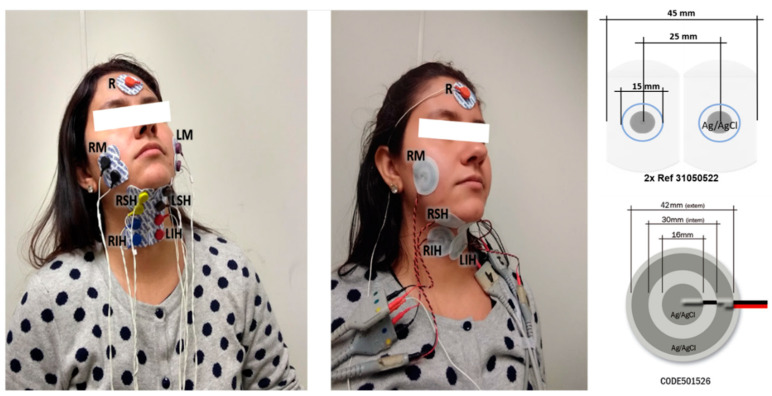
Electrode set-up for signal acquisition of the swallowing muscles with conventional disc electrodes (**left**) and concentric ring electrodes (**middle**); scheme and dimensions of electrodes (**right**). R reference; RM and LM, right and left masseters; RSH and LSH, right and left suprahyoid muscles; RIH and LIH, right and left infrahyoid muscles.

The myoelectrical activity of the muscle group was sensed by pairs of conventional disposable Ag/AgCl electrodes (Ref. 31050522, Covidien, Dublin, Ireland) that were 15 mm in diameter in the gel area with an inter-electrode distance of 25 mm, and CREs (CODE501526, Spes Medica, Battipaglia, Italy) made up of a central disc (16 mm diameter) and an external ring (28 mm and 42 mm inner and outer diameters). This disposable auto-adhesive and flexible pre-gelled Ag/Ag/Cl CRE was chosen because wet electrodes perform better than dry ones in short-term bioelectric recordings [35,36]. A disposable reference electrode (Ag/AgCl Ref. 2228, 3M, Sao Paulo, MN, USA) was placed on the forehead. Figure 2 shows the location of the conventional disc and CREs used in this study on the right and left masseters (RM and LM), right and left suprahyoid muscles (RSH and LSH), and on the right and left infrahyoid muscles (RIH and LIH). Gentle local abrasion (Nuprep, Weaver and Company, Aurora, CO, USA) of the skin and cleaning with isopropyl alcohol was performed before electrode placement to improve the skin-electrode impedance.

The sEMG signals were differentially amplified (gain 1000), band-pass (1–1000 Hz) and power line (50 Hz) filtered with commercial bioamplifiers (Grass Technologies P511, AstroNova Inc., West Warwick, RI, USA). Signals were acquired at a 5000 Hz sampling frequency with a DAQ device (USB NI-6229 BNC National Instrument, Austin, TX, USA). A camera was positioned diagonal to each participant and every swallowing task was video-recorded frame by frame and synchronized with the sEMG recordings by means of a custom Labview program.

The signals and video were recorded to characterize the activity while swallowing three different types of bolus: saliva, 10 mL of water and 10 mL of liquid yogurt. These three consistencies have been used by other authors to evaluate penetration/aspiration in dysphagia [37]. Liquid and yogurt were delivered to the oral cavity through a cup holding 1.5 oz. The seated subject was instructed to remain motionless in a relaxed position without swallowing for 5 s to obtain a reference for background activity. They were then asked to swallow the bolus as naturally as possible. The experiments were carried out first with CDEs, after which the electrodes were removed, the skin was cleaned and the CREs were placed for the second set of measurements.

### 2.3. Signal Analysis

As we were focused on the assessment of saliva and liquid boluses disregarding clenching or mastication, only the infrahyiod and suprahyiod muscle groups were evaluated. These groups are activated at the end of the oral phase and during the pharyngeal swallowing phase. The onset of the pharyngeal phase was verified on the recorded video.

Offline analysis was performed on a custom program (Matlab, MathWorks, Inc., v. R2019b, Natick, MA, USA). Two filter setups were used: to compute frequency domain features and following the SENIAM (Surface ElectroMyoGraphy for the Non-Invasive Assessment of Muscles) recommendations, the signals were filtered by a fifth-order Butterworth filter with band-pass between 10 and 500 Hz [38]; a narrower band-pass filter (between 25 and 400 Hz) [39] was applied to improve the signal quality before computing time domain features. The sEMG signals were then segmented using a semi-automatic program, which allowed for the simultaneous filtering and plotting the four acquisition channels. Only the segments without motion artifacts were used as the program allowed the digital annotation of time-stamps based on cursors controlled by the user. An expert manually selected the following segment types, which were later checked by a second expert.

**Figure 3:**
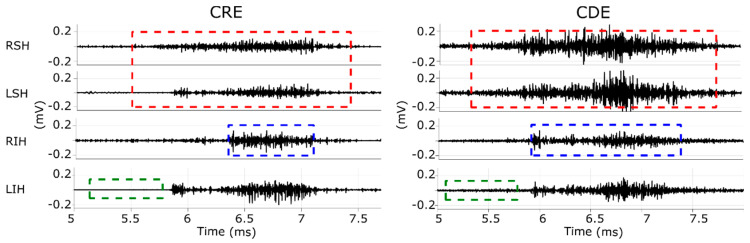
Example of sEMG signals recorded by bipolar CDEs (**right column**) and with CRE (**left column**) while swallowing water. RSH and LSH, right and left suprahyoid muscles; RIH and LIH, right and left infrahyoid muscles. Red-dashed squares in RLH and LSH mark segments used for correlation analysis. Blue-dashed squares in RIH mark a muscular contraction and green-dashed squares in LIH mark a background segment.

- Muscular contraction (burst): is comprise of the onset and offset time of each muscular activation executed by the participant during the swallowing tasks. RIH in Figure 3 shows an example of the segmentation of muscular contraction of the acquired signal from the right infrahyoid muscle with a blue-dashed box. Video recordings were used to verify that activation segments coincided with the swallowing task.
- Background noise: refers to a 0.5 s signal segment without any muscle activity, such as the one shown in a green-dashed box in LIH in Figure 3. The time intervals of these segments were the same for all the recorded channels and were generally extracted from the five-second interval before executing the swallowing task. Video recordings confirmed there was no swallowing activity in these segments.
- Correlation activity segment: refers to the bilateral muscular activity of the suprahyoid and infrahyoid muscles including approximately 0.5 s of basal activity prior to and after the swallowing process. The segments from each muscular group were saved in pairs for muscular correlation analysis. The red-dashed box in RSH and LSH in Figure 3 shows an example of this segment of the suprahyoid muscle’s signals during the pharyngeal phase. Video recordings were used to confirm that the segment was associated with the swallowing task and not with head or neck movements.

Three time domain and three frequency domain features were computed from every EMG contraction segment in order to analyze the differences in amplitude and frequency of the signals acquired by CREs and CDEs. Several previous studies used these features in sEMG recordings of the muscles used to swallow [11,40,41].

- Root mean square (RMS) is equivalent to the square root of the variance of the signal with zero mean and is a typical measure used to assess signal power in time domain.
  (1) $$\mathrm{RMS}=\sqrt{\frac{1}{N}\overset{N}{\underset{i}{\sum }}s_{i}^{2}}$$
  where $s_{i}$ is the sample i of the signal s, and N  is the number of samples.
- Signal-to-noise ratio (SNR) is computed as the ratio of the energy of the target signal (sEMG burst in this case) and background noise; it is usually given in decibels (dB) and gives information about signal quality.
  (2) $$\mathrm{SNR}=20\log\left(\frac{{\mathrm{RMS}}_{\mathrm{burst}}}{{\mathrm{RMS}}_{\mathrm{noise}}}\right)$$
  where ${\mathrm{RMS}}_{\mathrm{burst}}\;$ and $\;{\mathrm{RMS}}_{\mathrm{noise}}\;$ are the RMS computed in a segment corresponding to muscular contraction and background noise, respectively.
- Signal-to-motion ratio (SMR) gives information about the level of the motion artifacts in the signal. It was computed using the whole recorded signal according to the description given by [42]
  (3) $$\mathrm{SMR}=10\log\left(\frac{\sum_{k\in A}P_{k}}{\sum_{k\in B}P_{k}}\right)$$
  (4) $$A={\{P}_{k}\;|\;k\;\in \left[0,\frac{f_{s}}{2}\right]\}$$
  (5) $$B=\{P_{k}\;|k<20\;\mathrm{Hz}\;\wedge P_{k}>f\left(\alpha \right)\}$$
  where $P_{k}\;$ is the power to the k frequency in the power spectral density (PSD) and f(α) is a straight line between cero and the highest mean power density, $f_{s}$ is the sampling frequency: 5 kHz.
- Zero-crossing (ZC) provides indirect information about the time domain of the signal frequency. It indicates the number of zero-crossings with amplitudes greater than a threshold to avoid low voltage fluctuations and background noise [43]. In this work, this threshold (*Th*) was set to 3 times the standard deviation of the background noise segment.
  (6) $$\mathrm{ZC}=\overset{N-1}{\underset{i=1}{\sum }}\left[\emptyset \left(s_{i},s_{i+1}\right)\right]$$
  (7) $$\emptyset \left(x\right)=\{\begin{array}{c} 1\;if\;C_{1}<0\;\wedge \;C_{2}\geq Th\;\\ 0\;otherwise\; \end{array}$$
- Median frequency (MF) is the frequency below and above which 50% of the total power of the PSD lies.
  (8) $$\mathrm{MF}=\overset{\mathrm{MF}}{\underset{k=0}{\sum }}P_{k}=\overset{f_{s}/2}{\underset{k=\mathrm{MF}}{\sum }}P_{k}=\frac{1}{2}\overset{f_{s}/2}{\underset{k=0}{\sum }}P_{k}$$
- Bandwidth (BW) is the difference between the upper and lower frequencies of a signal where its spectral power is concentrated. In this work the upper and lower limits were computed considering a power tail of 5%.
  (9) $$\mathrm{BW}=F_{95\mathrm{th}}\left(\mathrm{PSD}\right)-F_{5\mathrm{th}}\left(\mathrm{PSD}\right)$$
  where $F_{95\mathrm{th}}\;$ and $\;F_{5\mathrm{th}}$ are the 95th and 5th percentiles of the PSD, respectively.

To mitigate the effects of noise, MF and BW were computed from the difference between the PSD of the burst and background noise segments. PSDs were obtained by the Welch method (window size of 1024 samples with 50% overlap).

Crosstalk and synchronization between pairs of bilateral muscles were assessed by the Pearson correlation between sEMG signals from both sides.

(10) $$\mathrm{correlation}=\frac{\sum_{i}^{N}\left(x_{i}-\;\bar{x}\right)\left(y_{i}-\;\bar{y}\right)}{\sum_{i}^{N}{\left(x_{i}-\;\bar{x}\right)}^{2}\sum_{i}^{N}{\left(y_{i}-\;\bar{y}\right)}^{2}}$$

where $x_{i}\;$ and $x_{y}$ are the signal samples from left and right sEMG, $\;\bar{x}\;$ and $\;\bar{y}\;$ are their respective means. 

Two schemes were applied, first, the correlation was computed using the raw sEMG signals (after band-pass filtering), after which the correlation was computed from the sEMG envelope. The envelope was obtained by a moving average filter with 200 samples applied to the rectified sEMG signal. 

### 2.4. Statistical Analysis

The results for the different features followed a non-normal distribution (Shapiro–Wilk test, *p* < 0.05). As a two-sided Wilcoxon signed-rank test for pairwise comparison showed no statistically significant difference (*p* > 0.05) between sEMG parameters recorded from the left and right sides of any muscle, electrode configuration or bolus, the sEMG parameters from both sides were combined in the subsequent analyses to facilitate the comparison of electrode configurations and provide greater statistical power. A two-sided Wilcoxon signed-rank test was used to assess significant differences in the pairwise comparison of parameters between CDE vs. CRE (configuration comparison) and suprahyoid vs. infrahyoid (muscle comparison) for each type of bolus (saliva, water, yogurt). A significance level of *p* = 0.05 was established for all the statistical tests performed on MATLAB (MathWorks, Inc., v. R2019b, Natick, MA, USA).

## 3. Results

**Figure 4:**
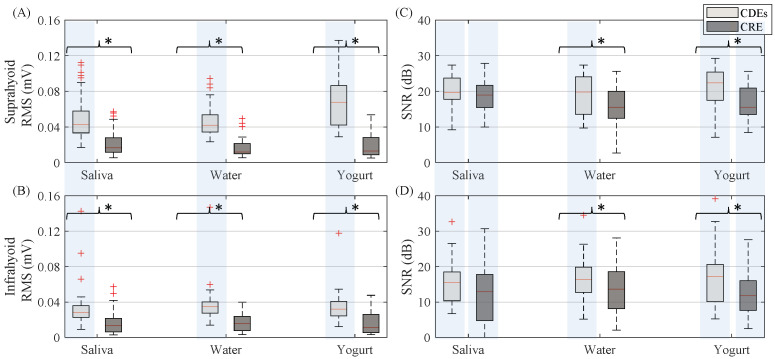
Box-whisker plots of RMS and signal-to-noise ratio of sEMG activity estimated from both suprahyoid and infrahyoid muscles recorded with CDEs and CREs swallowing saliva, water and yogurt. (**A**) Suprahyoid RMS. (**B**) Infrahyoid RMS. (**C**) Suprahyoid SNR. (**D**) Infrahyoid SNR. Statistically significant differences (*p* < 0.05) between electrode configurations are shown with an asterisk (*), between muscles with a shaded vertical stripe.

Figure 4 shows the RMS and signal-to-noise ratio of sEMG activity from CDEs and CRE configurations of the suprahyoid and infrahyoid muscles while swallowing saliva, water and yogurt. The CDEs sEMG RMS was significantly higher than that obtained from the concentric configuration (see also left and right panels in Figure 3), regardless of bolus and muscle type. The suprahyoid RMS was significantly higher than the infrahyoid for CDEs recordings, in contrast to the CREs with no noticeable difference between the muscles. The sEMG signal quality during swallowing was good, and obtained a median signal-to-noise ratio between 10 and 20 dB. Conventional recording obtained a better signal-to-noise ratio than those obtained from concentric ring electrodes for all bolus and muscle types, with a statistically significant difference for water and yogurt bolus from both the suprahyoid and infrahyoid muscles. Comparing the muscle groups, the suprahyoid signal-to-noise ratio was significantly higher than that from the infrahyoid in all cases except for the concentric configuration when swallowing water.

**Figure 5:**
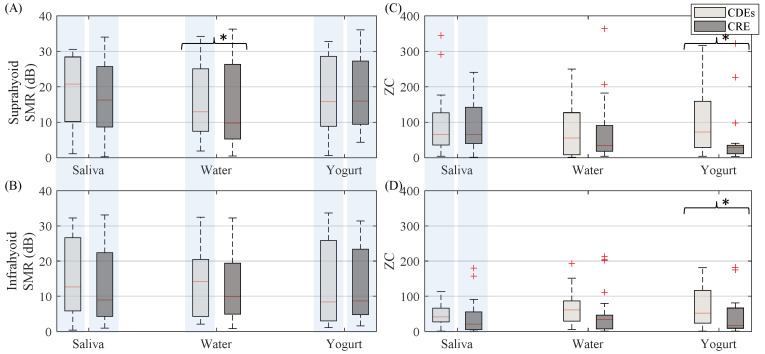
Box-whisker plots of SMR and zero-crossing of sEMG activity estimated from both suprahyoid and infrahyoid muscles recorded by CDEs and CREs when swallowing saliva, water and yogurt. (**A**) Suprahyoid SMR. (**B**) Infrahyoid SMR. (**C**) Suprahyoid zero-crossing. (**D**) Infrahyoid zero-crossing. Statistically significant differences (*p* < 0.05) between electrodes are shown with an asterisk (*), between muscles with a shaded vertical stripe.

It can be seen in Figure 5 that the SMR was similar for both electrode configuration with no remarkable differences when swallowing yogurt and it was slightly higher for CDEs when swallowing saliva. The only statistically significant difference between CDEs and CRE was found in the suprahyoid muscle when swallowing water. Both electrode configurations obtained significantly higher SMR values from the suprahyoid than the infrahyoid. 

In general, (see Figure 5) the CDEs sEMG zero-crossing was higher than that of the CRE, except when swallowing saliva in the suprahyoid, although a statistically significant difference was only obtained for the yogurt bolus from both the suprahyoid and infrahyoid. The sEMG activity in both configurations recorded significantly higher ZC from the suprahyoid than the infrahyoid when swallowing saliva, but not when swallowing water and yogurt.

**Figure 6:**
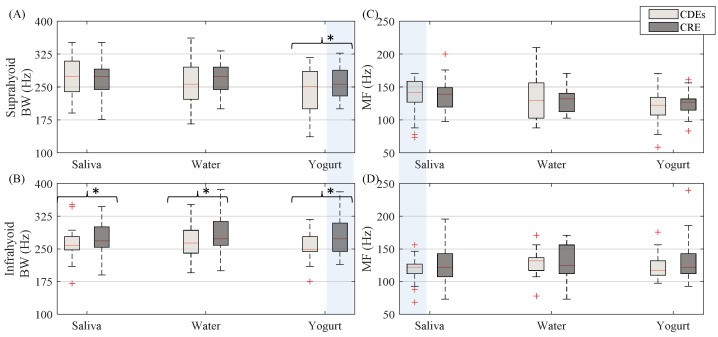
Box-whisker plots of sEMG bandwidth and median frequency estimated from both suprahyoid and infrahyoid muscles recorded with CDEs and CREs while swallowing saliva, water and yogurt. (**A**) Suprahyoid bandwidth. (**B**) Infrahyoid bandwidth. (**C**) Suprahyoid median frequency. (**D**) Infrahyoid median frequency. Statistically significant differences (*p* < 0.05) between electrode configurations are shown with an asterisk (*), between muscles with a shaded vertical stripe.

In the spectral domain, the CRE sEMG signal bandwidth (see Figure 6) was generally wider than that of conventional bipolar recordings with disc electrodes. This difference was statistically significant for yogurt in the suprahyoid and all types of bolus for the infrahyoid. Comparing the muscle groups, no statistical difference was obtained for the sEMG signal bandwidth between the suprahyoid and infrahyoid except when swallowing yogurt with the CRE. There was no noticeable difference between electrode configurations for median frequency (see Figure 6) for all muscle and bolus types, or between suprahyoid and infrahyoid except for swallowing saliva from CDEs sEMG recordings.

**Figure 7:**
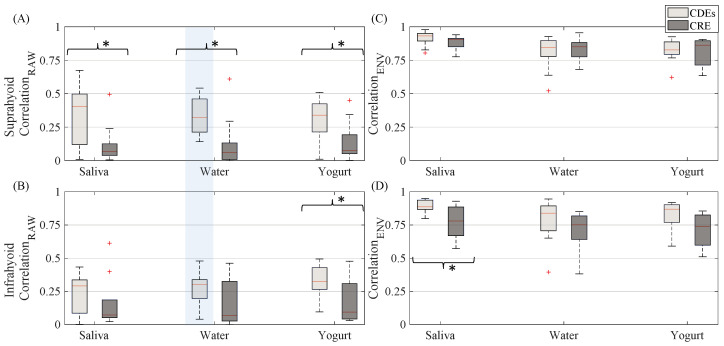
Box-whisker plots of correlation between EMG signals recorded from left and right sides estimated from both suprahyoid and infrahyoid recorded by CDEs and CREs while swallowing saliva, water and yogurt. (**A**) Correlation obtained from raw suprahyoid signal. (**B**) Correlation obtained from raw infrahyoid signal. (**C**) Correlation obtained from envelope suprahyoid signal. (**D**) Correlation obtained from infrahyoid envelope signal. Statistically significant differences (*p* < 0.05) between electrode configurations are shown by an asterisk (*), between muscles with a shaded vertical stripe.

The sEMG Pearson correlation of left and right-side muscles computed for both the raw and envelope signal is shown in Figure 7. The correlation estimated from the raw signal was lower than that obtained from the sEMG envelope signal. The CDEs sEMG usually obtained a higher raw signal correlation than the CRE configuration, as can be seen in Figure 3 in which RSH is more like the LSH (and RIH like LIH), in CDEs than CRE recordings. This difference in the correlation was statistically significant for all bolus types in the suprahyoid and for yogurt only in the infrahyoid. The correlation coefficient of the CDEs sEMG envelope signal was slightly higher than that obtained from the concentric configuration for infrahyoid, although the statistical difference was only significant for saliva. However, the electrode configuration did not affect the envelope signal’s correlation coefficient in the suprahyoid. Comparing the muscle groups, no difference was found in the correlation of raw or envelope signals regardless of electrode configuration and bolus type except for raw bipolar sEMG when swallowing water.

## 4. Discussion

In this work, a protocol was drawn up for recording muscle activity related to swallowing using concentric electrodes and conventional disc electrodes in a bipolar configuration—this is the first time that the CRE has been used for this purpose. The signals were characterized by various parameters typically used for sEMG signals from swallowing muscles to assess the possible effects, advantages and disadvantages of CRE versus CDEs recordings. Possible differences between the supra and infrahyoid muscle parameters were also analyzed to assess the performance of both electrode configurations.

Laplacian recordings by CRE have a different spatial transfer function to bipolar CDEs recordings [13]. CRE sensitivity to dipole sources at a distance from the electrode decreases faster than with disc electrodes in a bipolar configuration [13,20], which leads to enhanced spatial resolution and a smaller CRE half-sensitivity volume [44]. The CRE transfer function also reduces the low-pass filtering of signals picked up on the body surface associated with the blurring effect of the body volume conductor [45,46]. CREs also have axial symmetry, which alleviates electrode-to-fiber orientation issues [19] and makes their placement easier, especially important when fiber orientation is not easy to identify, as in the case of the swallowing muscles. 

The smaller sensing volume means fewer recordings from the muscle cells, which was responsible for the lower power (RMS) of the swallowing signals recorded by CRE in this study. Similar results have been reported when comparing CRE and CDEs recordings of other bioelectrical signals such as the electrocardiogram [47], electroenterogram [28] and electrohysterogram [25,26]. The smaller signal power can also lead to a poorer signal-to-noise ratio if the noise power is not reduced at a similar rate. As can be seen in Figure 4, differences in sEMG SNR recorded by CRE and CDEs are smaller than in RMS (also smaller *p*-values, not shown), which points to a greater relative reduction of noise. In fact, a statistical analysis of RMS from background noise segments (not shown) revealed that it was significantly smaller (*p* < 0.05) for CRE in all boluses for both supra and infrahyoid muscles. This led to no significant differences in SNR when swallowing saliva; however, the CRE SNR was still significantly smaller than those of CDEs for water and yogurt. CDEs and CRE obtained similar results for the presence of motion artifacts in sEMG recordings, with no significant differences in SMR except in the suprahyoid when swallowing water. Different factors affect signal quality and its characterization by SNR and SMR. In this study, SNR was estimated as the ratio of burst and basal segment power. This means that crosstalk from other muscles activated during swallowing would also be (incorrectly) considered as a contribution to “target signal” power. Similarly, greater power in the muscular bandwidth due to the contributions of other muscles would also affect SMR. Nonetheless, it is also clear that the electronic instrumentation in the signal conditioning and acquisition makes a “constant” contribution to noise that cannot be reduced by the electrode configuration. Other aspects such as muscle size, depth, thickness and composition of the layers between the electrode and target activity sources, etc., can affect the signal quality of both electrode configurations. No differences were obtained in forearm sEMG SNR [48] or from the uterine muscle [26], while they were lower for CREs in ECG [49] and biceps brachii sEMG [50]. No SMR results have been previously reported in CRE vs. CDEs sEMG comparisons.

With regard to the crosstalk issue, it is well known that sEMG measurements from swallowing muscle can be severely affected by the activity of the surrounding muscles [9,14,31]. Recordings of supra and infrahyoid muscle activity on each side can also pick up activity from the muscle on the opposite side or platysma, among others. The cross-correlation function has been traditionally used to determine the amount of common signal present that are in the recording channels [51]. In this study it was computed from raw sEMG signals to assess the effect of CRE’s enhanced spatial resolution on crosstalk. The results showed that correlation was significantly smaller in CRE sEMG signals than in CDEs recordings. Enhancement depends on the depth of the bioelectric dipoles [13], and as obtained in the present study, its effects are more pronounced in muscles near the surface, i.e., differences were greater for the suprahyoid muscle, which is nearer the surface than the infrahyoid, which is deeper and covered by more fatty tissue and looser skin. It could be thought that the smaller correlation with CREs could be attributed not to reduced crosstalk, but to less intense swallowing. However, when computing the cross-correlation of the envelopes of sEMG signals, which are more representative of the mechanical swallowing muscle activity, both electrode configurations showed similar results. The values were typically above 0.75, indicating good swallow synchronization on both sides, as could be expected in healthy subjects. The significant reduction of cross-talk would help CREs to perform more specific evaluations of the different swallowing muscles, and thereby, better characterize possible disorders. Facial, oral or pharyngeal asymmetric movements and structures due to weakness or poor neuromuscular coordination suggest oropharyngeal dysphagia [52], which would be easier to identify with reduced crosstalk.

Regarding the blurring effect of the volume conductor, which affects the spectral content of original signals when detected on the body surface, concentric electrodes have been shown to reduce the spatial filtering effects of spectral components in other sEMG signals [26,50]. In this study we obtained larger bandwidths for concentric recordings than for the conventional disc, especially in the suprahyoid region. This means that the sEMG activity for the swallowing muscle can be picked up and studied in a wider range of frequencies, closer to one of the original signals (invasive recording [53]) by CREs. This would provide more information on the activated swallowing muscle fibers than the CDEs configuration. In the present work with healthy volunteers no significant difference was obtained in the median frequency, which suggests that this bandwidth enlargement was somehow symmetrical. On the other hand, the ZC, which characterizes the signal frequency in the temporal domain, showed slightly higher values for the CDEs (significant for yogurt recordings). This is possibly due to this parameter only counting the zero-crossings of signals with a greater amplitude than the threshold (based on the noise level). Since the SNR was larger for CDE recordings (especially with yogurt), a greater number of polarity changes beyond that threshold could be expected even when similar median frequency values were obtained.

The results of the muscle comparison (supra vs. infrahyoid) in healthy volunteers showed similar results for both electrode configurations including greater signal quality (SNR, SMR) for suprahyoid signals in general, similar frequency content (BW, MF and zero-crossing, except for saliva) and coupling (cross-correlation) between the sides of the muscle. The only aspect that was noticeably different in the muscle comparison for both electrode configurations was in the signal amplitude (RMS), where significantly higher values for supra were recorded with CDEs, while no differences were found in the CRE recordings. These results are consistent with previous findings in the literature, which showed that using standard electrodes resulted in higher amplitude in suprahyoid signals during muscle activity [39] and significantly higher background activity in the infrahyoid affecting the SNR [54]. The “usual” poorer quality of signals from the infrahyoid muscle together with the lower SNR of CRE vs. CDEs may have been responsible for the lack of significant RMS differences between the supra and infrahyoid recordings with the CRE configuration.

Certain limitations of the study should be noted, especially with regard to the composition of the database, there was only a small number of young and healthy subjects (21) and they were mostly women (76%). However, according to some authors [55,56] no significant bias can be expected due to gender imbalance. After assessing the capability and potential of CRE in sEMG recordings of muscles related to swallowing in the present work, the population will be expanded in our future work to other age ranges including pathological cases with a better balance between the sexes. It will also evaluate whether CREs with these or other parameters of interest (such as time lags in muscle activation and/or deactivation, asymmetries in characteristic signal parameters or side desynchronization, signal complexity, etc.) allow better discrimination of controls and patients, with an assessment of the progress of the disease and possible treatments. The influence of the type of bolus swallowed will also be evaluated in greater detail, including other volumes and consistencies.

## 5. Conclusions

This work showed the feasibility of capturing the myoelectric activity of the swallowing muscles by a CRE and compared the results with sEMG recordings made with CDEs.

sEMG analysis of the swallowing muscles and dysphagia diagnosis and characterization can benefit from the use of the concentric electrode configuration compared to conventional bipolar disc electrodes. The advantages include enhanced spatial selectivity, reduced crosstalk from adjacent muscles and a wider range of EMG components. Placing the CRE on the body is also easier since it does not have to be oriented with the muscle fibers, unlike the bipolar configuration with CDE. This is particularly relevant in swallowing muscles in which visually determining fiber orientation can be challenging. However, it is recommended that technical changes be adopted in the future to ensure that the CRE’s lower signal amplitude does not significantly affect its quality.

CREs have great potential for improving clinical monitoring and evaluation of swallowing muscle activity. Further work on subjects with a wider range of ages and pathological cases will be necessary to further assess the advantages of CREs for sEMG recordings in dysphagia monitoring and diagnosis.
